# Prolonged post-hyperventilation apnea in two young adults with hyperventilation syndrome

**DOI:** 10.1186/1751-0759-7-9

**Published:** 2013-04-17

**Authors:** Takao Munemoto, Akinori Masuda, Nobuatsu Nagai, Muneki Tanaka, Soejima Yuji

**Affiliations:** 1 Department of Domestic Science, Kagoshima Women’s College, 6-9 kourai-chou, Kagoshima, 890-8520, Japan; 2 Masuda Clinic,Idehara-BLDG 6F, 1-30 Yamanokuchi-chou, Kagoshima, 892-0844, Japan; 3 Nogami Hospital, 1-4-1 Komatsubara, Kagoshima, 891-0114, Japan; 4 Yoshimura Hospital, Kagoshima, Dairyu-chou, 892-0805, Japan; 5 Department of Sports and Life Science, Faculty of Physical Education, National Institute of Fitness and Sports in Kanoya, 1 hakusui-cho, Kanoya, 891-2393, Japan

**Keywords:** Post-hyperventilation apnea, PHA, Hyperventilation syndrome, HVS, Hypocapnia, Hypoxemia

## Abstract

**Background:**

The prognosis of hyperventilation syndrome (HVS) is generally good. However, it is important to proceed with care when treating HVS because cases of death following hyperventilation have been reported. This paper was done to demonstrate the clinical risk of post-hyperventilation apnea (PHA) in patients with HVS.

**Case presentation:**

We treated two patients with HVS who suffered from PHA. The first, a 21-year-old woman, had a maximum duration of PHA of about 3.5 minutes and an oxygen saturation (SpO_2_) level of 60%. The second patient, a 22-year-old woman, had a maximum duration of PHA of about 3 minutes and an SpO_2_ level of 66%. Both patients had loss of consciousness and cyanosis. Because there is no widely accepted regimen for treating patients with prolonged PHA related to HVS, we administered artificial ventilation to both patients using a bag mask and both recovered without any after effects.

**Conclusion:**

These cases show that some patients with HVS develop prolonged PHA or severe hypoxia, which has been shown to lead to death in some cases. Proper treatment must be given to patients with HVS who develop PHA to protect against this possibility. If prolonged PHA or severe hypoxemia arises, respiratory assistance using a bag mask must be done immediately.

## Background

Hyperventilation syndrome (HVS) is characterized by functional hyperventilation attacks with no underlying organic abnormality. In Japan, 508 patients with acute HVS were reported to range in age from 5–85 years, and acute HVS was particularly prevalent among women in their late teens and among men in their twenties [1]. The male-to-female ratio is 3 to 7 [1]. HVS is also related to psychosomatic stressors. HVS patients show various clinical symptoms such as anxiety, dyspnea, hypocapnia, tetany, and unconsciousness.

Traditionally, it has been considered that hyperventilation attacks spontaneously disappear and that the paper-bag method or the administration of anxiolytic agents leads to the disappearance of such attacks regardless of their severity. However, there is little evidence regarding the use of the paper-bag method for HVS and the incidence of death, severe hypoxia, or myocardial infarction in association with the paper-bag method [2]. Recently, the paper-bag method was not recommended for the treatment of patients with HVS [2].

We have experienced several patients with HVS who had post-hyperventilation apnea(PHA). PHA is apnea that follows hyperventilation due to HVS or another cause.

In most cases, the patient spontaneously recovered from PHA within one minute without any clinical problems. Moreover, we recently encountered two patients with HVS in whom PHA persisted with cyanosis, hypoxemia, and loss of consciousness for more than three minutes. We were confronted with difficult decisions as to whether or not to perform cardiopulmonary resuscitation (CPR) on these patients.

Previously, Haldane and Douglas reported PHA in humans [3,4]. Some studies have indicated experimental PHA in healthy adults [5,6]. According to several studies, PHA is frequent in patients with central nervous abnormalities [7,8], can be complicated by lung disease (pulmonary emphysema, bronchial asthma) [9,10], and led to the death of one patient [9]. Few studies have reported PHA related to HVS. To our knowledge, only Inagaki [11], MacDonald [12], and Chin [13] reported PHA with hypoxemia related to HVS. However, aside from MacDonald’s case, there are few previous case reports of HVS patients who were rescued from PHA after having long term PHA accompanied by cyanosis, hypoxemia, and loss of consciousness. Here we present two patients with HVS who developed prolonged PHA. In analyzing these cases, the risk of prolonged PHA and the possible treatment for hyperventilation attack were examined. The aim of this report is to demonstrate the development and clinical risk of PHA with severe hypoxemia in patients with HVS.

## Case presentation

### Patient 1

The patient was a 21-year-old woman. At the age of 18 years, she suffered a hyperventilation attack following stressors at her nursing school. Her strongest stressor was practicing as a nurse at the hospital. She was subsequently hospitalized several times for frequent hyperventilation attacks. There were no organic abnormalities according to several medical examinations including a blood test, chest X-ray, electrocardiogram, electroencephalogram and head CT. She was diagnosed with HVS. Her hyperventilation attacks gradually decreased in response to the paper-bag method or anxiolytic agents administered during hospitalization. For approximately one year after being discharged from the hospital, no attacks occurred. However, the hyperventilation attacks recurred when triggered by acute tonsillitis. Because of the frequent attacks she was referred to our hospital to undergo a regimen of psychotherapy and fasting therapy [14,15], which is effective for various stress-related diseases. Immediately on hospitalization, she developed frequent hyperventilation attacks.

### Patient 2

The patient was a 22-year-old woman. At the age of 21 years, she suffered from affective disorder following sexual harassment in her office. Her symptoms were relieved by antidepressants and counseling. However, she was admitted to our hospital for relapse, which might have been caused by her stopping taking the medication without consulting the attending physician. After her admission, she began to improve. About one month after admission, a visit from one of her coworkers reminded her of several episodes of sexual harassment, and her emotional state became unstable. She then developed hyperventilation attacks for the first time.

### Clinical profiles of the patients

There was no medical/family history of bronchial asthma or epilepsy for either patient. On admission, the body mass index (BMI) values of patients 1 and 2 were 20.1 and 17.8, respectively. We performed several medical examinations, such as blood tests, chest X-ray, echocardiogram, electrocardiogram, and cephalic CT, to rule out organic diseases that could cause hyperventilation. There were no abnormalities in the blood test results of either patient(WBC: 4800/μL and 5200/μL, Hb: 13.1 g/dL and 11.3 g/dL, platelet: 20.1 × 10^4^/μL and 28.9 × 10^4^/μL, AST: 23 IU/L and 18, ALT: 13 IU/L and 17 IU/L,s-AMY: 66 IU/L and 97 IU/L,CRP: none and o.1 mg/dL, creatinine: 0.7 mg/dL and o.9 mg/dL, Na: 143 mEq/L and 141 mEq/L, K: 3.9 mEq/L and 4.3 mEq/L, fT_4_: 1.2 ng/dL and i.0 ng/dL, fT_3_: 1.2 pg/dL and 3.3 pg/dL,TSH: 0.41 μIU/mL and 1.08 μIU/mL). There were no abnormal findings in the chest X-ray, electrocardiogram, echocardiogram, or cephalic CT, so lung, heart and brain disease were ruled out. A hyperventilation attack was identified based on respiration that obviously exceeded the usual breathing rate (about 12 to 20 breaths per minute) and clinical symptoms such as tetany. A physician used a stethoscope to confirm that there were no airway stenosis sounds or abnormal alveolar sounds. We could not analyze arterial blood-gas to prove hypocapnia because the hospital did not possess the device needed to perform this analysis. Neither patient presented with hypoxemia during the hyperventilation attacks or normal respiration. Therefore, the possibility of a hyperventilation attack being caused by other disorders was ruled out. Although the arterial PCO_2_ level was not measured for either patient, the clinical presentation left little doubt that over-breathing resulted in HVS. To examine the psychological aspects, the Minnesota multiphasic personality inventory(MMPI)was performed. The MMPI showed high scores of Hs: 30, D: 48 and Hy: 33 for Patient 1 and Sc: 54, Pt: 49 and D:35 for Patient 2. These data suggest a depressive state for both patients, namely anxiety about her physical symptoms by Patient 1 and unreasonable fears or anxiety and obsessions by Patient 2. In both patients, some stressors were present prior to the hyperventilation attacks. In case 1, acute tonsillitis induced the hyperventilation attacks, which occurred frequently and increased her anxiety level. Immediately on hospitalization, she developed frequent hyperventilation attacks before fasting therapy [14,15] was begun. She agreed to undergo fasting therapy but had considerable anxiety over receiving the therapy. Hospitalization and anxiety about the fasting therapy became the stressors and triggers of her hyperventilation attacks after admission to the hospital. In case 2, a co-worker’s visit to her in the hospital evoked memories of the sexual harassment in the office and induced hyperventilation attacks. Both cases were diagnosed with HVS because they had preceding stressors and no organic abnormalities that could cause hyperventilation attacks.

### Hospital course of PHA

**Figure 1:**
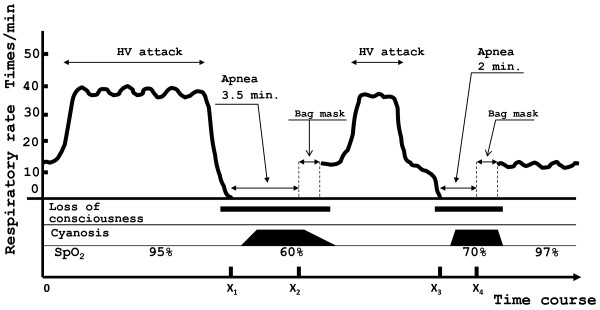
**Clinical course of apnea and HV attacks on fifth day after admission: Patient 1.** The patient presented a 3.5-minute episode of apnea after an HV attack with a minimum SpO_2_ of 60% and cyanosis of the lips and nail beds. The patient did not exhibit hypoxemia during the HV attack or during normal respiration. X_1_ and X_3_ represent the apneic starting time. *X*_2_ and X_4_ represent the time of the assisted ventilation by bag-mask. X_1_X_2_ and X_3_X_4_ are the lengths of the apneic times, which were 3.5 and 2 minutes, respectively. X_1_X_2_ and X_3_X_4_ were recorded, but times X_1,_*X*_2,_ X_3,_ and X_4_ were not recorded correctly. HV: hyperventilation.

Figure 1 shows a series of hyperventilation attacks by Patient 1 on the fifth day after admission. PHA persisted for 3.5 minutes, and oxygen saturation (SpO_2_) decreased to 60%. Cyanosis of the lips and nail beds developed. She did not respond to pain stimulation. Loss of consciousness was observed, and spontaneous respiration did not start again. Therefore, artificial respiration was initiated by the bag mask method. After a few minutes, spontaneous respiration started again. Then, an additional hyperventilation attack occurred, followed by apnea for two minutes. Simultaneously, cyanosis of the lips developed, and SpO_2_ decreased to 70%. Artificial respiration with a bag mask was performed, and after a few minutes, spontaneous respiration started again. The series of attacks ended.

**Figure 2:**
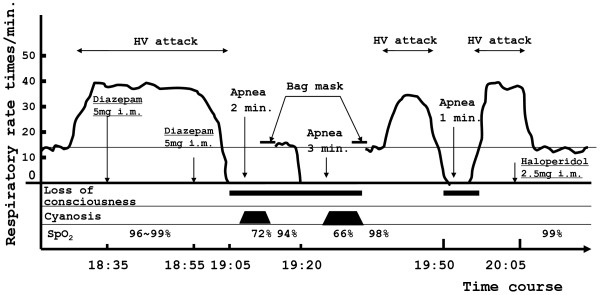
**Clinical course of apnea and HV attacks about one month after admission: Patient 2.** The patient presented a 3-minute episode of apnea after an HV attack with a minimum SpO_2_ of 66% and cyanosis of the lips. The patient did not show hypoxemia during the hyperventilation attack or during normal respiration. HV: hyperventilation, I.m.: intramuscular injection.

The course of PHA in Patient 2 is shown in Figure 2. After a 30-minute hyperventilation attack, apnea persisted for two minutes, causing the lips to become cyanotic and SpO_2_ to decrease to 72%. Spontaneous respiration re-started without treatment. However, after a few minutes, additional PHA for 3 minutes and cyanosis of the lips and face were observed. SpO_2_ was 66%. Even after three minutes, respiration did not start again, and artificial respiration with a bag mask was induced. A few minutes later, spontaneous respiration started again. Hyperventilation attacks then occurred repeatedly. To treat the initial hyperventilation attack, 10 mg diazepam was intramuscularly administered by dividing the dose into two boluses to prevent apnea. To treat the subsequent hyperventilation attack, 2.5 mg haloperidol was intramuscularly administered.

SpO_2_ was measured with a pulse oximeter (NIHON KOHDEN, SAT-2100 with finger probe). The physician measured the SpO_2_ of the patients several times during hyperventilation and normal breathing following hyperventilation, SpO_2_ and recorded minimum SpO_2_ were frequently measured during PHA. Because the pulse oximeter could not save the measured SpO_2_ data, not all of the data were saved.

The respiratory rate (RR) is defined as the number of breaths per minute. The RR was measured at least one time during hyperventilation. PHA was identified based on the absence of breath sounds at auscultation and the absence of wall motion of the chest. RR and length of PHA were measured by a nurse, following the physician’s directions. Loss of consciousness was determined based on the absence of response to painful stimuli to the sternum and the absence of response to calling. Cyanosis was determined by the physician’s inspection of the skin of the patients.

## Discussion

We experienced two patients with HVS who showed prolonged PHA with hypoxemia, cyanosis, and loss of consciousness. There have been many reports of PHA, but few clinical reports of prolonged PHA have been made. Though the risk of death is present for patients with HVS who present severe hypoxemia following prolonged PHA, no management strategy for patients with extended PHA has been established. In this report we demonstrated the treatment course of patients with HVS who had prolonged PHA with severe hypoxemia and discussed the mechanism, risk of death, and management of PHA.

### PHA and BHS

As described above, only a few studies have reported PHA related to HVS. Inagaki et al. and MacDonald et al. have reported PHA related to HVS during adolescence or young adulthood. Inagaki’s cases were a 17-year-old boy and a 21-year-old woman [11], whose presentations were reported as breath-holding spells (BHS). BHS in children is a disease entity resembling PHA, and a common disease in infants. BHS causes apnea, cyanosis, pale face, and loss of consciousness following crying induced by pain, anger and fear. The patient spontaneously recovers. BHS resembles PHA in terms of the development of apnea after hyperventilation. PHA and BHS differ in the age of onset. MacDonald’s case was a 14-year-old girl [12]. The mechanism of BHS is associated with various physiological (dysregulation of vagal nerve, anemia, etc.) and psychological (infant crying induced by pain, anger, and fear) factors [16,17]. One fatality was reported [18].

### Prolonged PHA and Hypoxemia

In Inagaki and MacDonald’s patients, cyanosis and hypoxemia were observed. PHA in Inagaki’s cases persisted for one to two minutes, resulting in spontaneous recovery [11]. PHA in MacDonald’s patient persisted for three minutes and showed a minimum PaO_2_ of 25 mmHg. Therefore, intratracheal intubation with an artificial respirator was performed for respiration control [12]. MacDonald’s case was reported as an example of a near-fatal instance of PHA. Chin reported hypoxemia and PHA in patients with HVS [13]. Three patients presented PHA, and their SaO_2_ levels were 48%, 63%, and 68%. The SaO_2_ of four patients ranged from 71% to 81%. The PaO_2_ of five patients ranged from 32 torr to 64 torr. In our patients, PHA persisted for three minutes or more in the presence of cyanosis, hypoxemia, and loss of consciousness (minimum SpO_2_: 60% and 66%, respectively). Immediately, artificial respiration with a bag mask was administered, and respiration recovery was soon achieved. Our cases were serious and presented long durations of PHA related to HVS.

### Mechanism of PHA

The phenomenon of PHA has been observed by many researchers. PHA has been reported to be due to a decreased PaCO_2_[3,19] and has been reported to be induced when PaCO_2_ was reduced to the threshold [20,21]. Hypocapnia caused by hyperventilation presents the condition of alkalosis. In alkalosis, reduced hydrogen ions [H^+^] acting on the chemoreceptors lead to the suppression of breathing [20].

Even if hypocapnia after hyperventilation is normalized, the stimuli to begin breathing do not immediately restart, because the threshold level of PaCO_2_ is elevated [22]. This seems likely to be associated with persisting hypocapnia in the brain, even when arterial and alveolar PaCO_2_ are normalized. Cummin et al. [23] demonstrated by a mathematical simulation that central chemoreceptor PCO_2_ recovered more slowly than arterial PaCO_2_. In addition, reduced vigilance after hyperventilation seems to be related to the appearance of PHA according to Mangin et al. [8]. They reported that post-hyperventilation sleep reduces central neural facilitations, allowing the lack of chemical stimulation to induce apnea [8]. These studies may explain the mechanism of PHA.

While the prolongation of PHA seems to be closely linked to the duration of a hyperventilation attack [24], Meah et al. reported that, conversely, the length of PHA was independent of the length of the hyperventilation attack [6]. The hypoxic ventilatory drive is attenuated at hypocapnia due to the hyperventilation attack [25,26], which might reduce breathing stimulation and elongate PHA even during hypoxia. This might worsen hypoxemia. Also, this is regarded as one of the mechanisms of post-hyperventilation hypoxemia. Other mechanisms of post-hyperventilation hypoxemia include a lack of oxygen intake during prolonged PHA. In addition, because the oxygen stores in the body are far lower than the carbon dioxide stores, a slow restoration of carbon dioxide after a hyperventilation attack would lead to hypoxemia more rapidly than PaCO_2_ would rise [27]. Furthermore, Nolan et al. demonstrated that post-hyperventilation hypoxemia was due to the alteration of pulmonary blood flow distribution, causing a drop in the V/Q ratio [28]. It was reported that post-hyperventilation hypoxemia might be dependent on the severity of the hypocapnia [29].

We thought that the prolongation of PHA of our patients might be due to several factors, such as the delay of PCO_2_ recovery in the central chemoreceptor, long hyperpnoea, and unconsciousness during PHA. As for post-hyperventilation hypoxemia with cyanosis, a lack of oxygen intake caused by long PHA, the attenuation of the hypoxic ventilator drive, a lower oxygen store, and alteration of the pulmonary blood flow distribution might have played an important role. In case 2, we cannot deny the possibility that diazepam might have participated in the respiratory depression to a certain extent.

### Drug prescription of Diazepam

Although diazepam has a risk of respiratory inhibition, we divided its administration into two boluses to prevent apnea. The 10 mg of total diazepam was not an overdose. We assume that the 10 mg of diazepam partially contributed to the apnea, but was not the only factor. Diazepam is a long-acting antianxiety sedative and can cause respiratory depression [30]. A standard dose of diazepam is considered to be 0.25 ~ 0.30 mg/kg (intravenous administration) [25,31]. The initial dose of diazepam administered to patient 2 was 5 mg (0.11 mg/kg), and a dose of 5 mg was added about 30 minutes later. The total dosage of 10 mg (0.22 mg/kg) was not excessive compared with the standard dosage. In addition, we administered the diazepam by intramuscular injection, which was expected to act gradually. We believe that our means of administration of diazepam was proper to prevent respiratory depression or apnea. However it is possible that the diazepam might have participated partially in the prolonged PHA of patient 2, because the effectiveness of diazepam varies greatly from person to person. However, we cannot identify the degree to which diazepam was a factor in the PHA. Therefore, diazepam should be chosen very carefully to prevent prolonged PHA and cyanosis in the treatment of hyperventilation attacks related to HVS.

### Risk of PHA

It remains to be clarified whether or not PHA related to HVS in humans can be fatal. However, some studies have identified related issues.

MacDonald reported a near-fatal case of PHA related to HVS [12]. The patient was successfully treated with intubation and ventilation. Chin et al. reported cases with HVS that presented long PHA and severe hypoxemia [13]. The treatments administered to the patients were not mentioned.

In experiments on animals, the potential lethal effect of PHA was reported by Mosso [32,33] when he hyperventilated nine dogs. After hyperventilation, five of the dogs were left entirely undisturbed to either die apneic or to recover unaided. Two dogs died. The degree of hypoxia observed was striking. At the beginning of their experiment, the arterial oxygen content was 14.8 volume percent and the arterial carbon dioxide content was 16.2 volume percent. At the end of eight minutes of apnea, the arterial oxygen content was zero, and the arterial carbon dioxide content was 21.7 volume percent. Based on the above findings, we cannot deny the possibility that PHA related to HVS can lead to a fatal outcome.

### Comment about the treatment of HVS

Although HVS is generally reversible and shows a good prognosis, its treatment should be administered with care, because post-hyperventilation apneic cases or other cases that resulted in death [2,34] have been reported. As mentioned above, there is a risk of death, especially in prolonged PHA related to HVS. We should assume that some patients with HVS could develop PHA or post-hyperventilation hypoxia with cyanosis. It is not clear whether PHA related to HVS can be fatal or not, but emergency treatment such as CPR should always be available considering the possibility of a fatal outcome. We propose that respiratory assistance by bag mask should be performed for critical situations in PHA with hypoxemia, while blood–gas analysis and the monitoring of SpO_2_ and other vital signs such as heart rate, blood pressure, etc., should be performed. It is desirable that a proper amount of oxygen be given to the patient, because hypoxemia is not good for the brain and hypoxemia at hypocapnia does not stimulate respiration. Also we should be very careful about using sedatives such as diazepam, which can cause respiratory depression, to stop a hyperventilation attack, because we cannot exclude the possibility that they will induce PHA. It is a traditional practice to treat hyperventilation attacks related to HVS by paper-bag rebreathing. However the paper-bag method seems to be a potentially risky procedure, as death, hypoxia and collapse have been reported to occur with paper-bag rebreathing [2]. Furthermore, the effectiveness of the paper-bag method is not clear. Thus, paper-bag rebreathing should not be used in hyperventilation attacks.

### Limitations of report

Although we believe that this report provides clinically important and useful information about PHA and HVS, we were not able to show sufficient objective data to evaluate the pulmonary and breathing functions. This report is retrospective and it was not possible to collect all necessary data. Particularly, it was unfortunate that we could not analyze the arterial blood-gas of patients in order to accurately evaluate PaCO_2_, PH, and PaO_2_, which demonstrate hypocapnia or hypoxemia. Also, continuous monitoring of the breathing rate and oxygen saturation to evaluate and examine the hyperpnoea and the PHA was not possible. These problems were encountered with regard to the diagnosis of HVS and to the study of the mechanism and treatment of PHA. However, the diagnosis of HVS was based on clinical findings and other medical examinations, and consequently we have no doubt that HVS was correctly diagnosed. To study PHA related with HVS, physicians should prepare a system of objective data collection to evaluate the pulmonary and breathing functions, because the opportunities to encounter PHA caused by HVS are limited.

## Conclusion

These cases show that some patients with HVS develop prolonged PHA or severe hypoxia, which is believed to be potentially risky based on its lethality in some cases. Proper emergency treatment should be available for patients with HVS who develop PHA. If prolonged PHA or severe hypoxemia arises, it is necessary to immediately perform respiratory assistance by bag mask as a precautionary measure.

### Consent

Written informed consent was obtained from both patients for the publication of these cases and any accompanying images. A copy of the written consent is available for review by the Editor-in-chief of this journal.

## Abbreviations

HVS: Hyperventilation syndrome; PHA: Post-hyperventilation apnea; BHS: Breath-holding syndrome; BMI: Body mass index; MMPI: Minnesota multiphasic personality inventory; SpO2: Oxygen saturation; PaO2: Partial pressure of oxygen in artery; PCO2: Partial pressure of carbon dioxide; PaCO2: Partial pressure of carbon dioxide in artery; V/Q: Ventilation/perfusion ratio; RR: Respiratory rate.

## Competing interests

The authors declare that they have no competing interests.

## Authors’ contribution

TM carried out the medical treatment and drafted the manuscript. NN and MT participated in the medical treatment. AM conceived of this report and helped to draft the manuscript. YS helped to draft the manuscript. All authors read and approved the final manuscript.
